# Mapping interdisciplinary collaboration in music education: analysis of models in higher education across North America, Europe, Oceania, and Asia

**DOI:** 10.3389/fpsyg.2023.1284193

**Published:** 2023-11-29

**Authors:** Guozhong Zhang, Jian Sun, Ying Sun

**Affiliations:** ^1^School of Music and Dance, Xihua University, Chengdu, Sichuan, China; ^2^School of Vocational Education, Tianjin University of Technology and Education, Tianjin, China

**Keywords:** interdisciplinary collaboration, comprehensive analysis, music education, higher education, innovation

## Abstract

Interdisciplinary collaboration is an emerging imperative in music education, but current understanding of global practices remains limited. Prior studies have focused narrowly on specific programs within limited geographic areas. However, there is minimal quantitative mapping of initiatives across institutions and regions. Contextual insights explaining regional variations are also scarce. Hence, this study aims to address these gaps by conducting a comprehensive analysis of interdisciplinary programs, partnerships, events, and publications across leading global music institutions using a mixed methods approach. The analysis reveals significant diversity in models and determinants of productivity across regions. For instance, while Europe leads in formal integration and research output, North America prioritizes technology-enabled innovation through media and emerging tools. Partnerships are ubiquitous but focus areas and curricular integration vary. The findings reveal significant diversity in interdisciplinary education practices and formats worldwide, implying a need for contextualized implementation aligned to institutional strengths rather than a one-size-fits-all approach. Therefore, as music education evolves, tailored interdisciplinary strategies that blend local priorities with global best practices are recommended to creatively nurture multifaceted skills and maximize potential for innovation. Thus, this research contributes an invaluable knowledge base to inform evidence-based, nimble policies and frameworks for cultivating cutting-edge, socially engaged musicians and ventures worldwide.

## 1 Introduction

Interdisciplinary education is an approach that aims to provide a more comprehensive understanding of complex topics by integrating information or methods from two or more academic disciplines (Spelt et al., 2009; Corbacho et al., 2021). Through interdisciplinary courses and projects, learners integrate relevant theories, methods, and findings from two or more fields which makes it not only complex but also challenging because it seeks to produce new knowledge by understanding complex problems (Golding, 2009; Spelt et al., 2009). This allows them to examine issues, solve problems or explore new questions in ways not possible through just one subject alone, enabling integrated understanding and transfer of learning across fields. Moreover, this helps to cultivate innovative, adaptive thinking skills needed to operate at the intersections of ideas from different domains (Corbacho et al., 2021). Thus, these attributes make interdisciplinarity unique and distinct from multidisciplinary in education, which refers to an approach that brings together knowledge and methods from multiple disciplines but does not necessarily integrate them (Lombardo, 2010). For instance, a music education program that combines music theory, history, and performance techniques to provide a comprehensive understanding of music is multidisciplinary whereas a research project or program that explores the intersection of music education and technology, incorporating elements of computer science, engineering, and musicology to develop innovative music teaching tools can be termed interdisciplinary (Hodges, 2003; Barneva et al., 2021; Zhang, 2022). Interdisciplinary education is mostly offered at advanced undergraduate or postgraduate levels as it remains crucial for undergraduate students to experience integrative learning environments that support an open perspective toward other disciplinary fields (Lyall et al., 2015).

What is more, interdisciplinarity has become an increasingly popular approach in various fields of study, including music education. The interdisciplinarity of music education considers the interconnections between musical and non-musical modes of thinking through various academic and creative works beyond just educational methods. An example of interdisciplinary music studies the incorporation of music technology and computer science to develop innovative tools for music composition and production. Others include the integration of music therapy and psychology to explore the therapeutic effects of music on mental health and the combination of musicology and cultural studies to examine the sociocultural influences on music production and consumption (Schei et al., 2013). This approach of interdisciplinarity is increasingly being embraced to foster multifaceted skills and drive innovation (Baroni, 2021). The growing emphasis on interdisciplinarity can be attributed to several factors; recognition of the significance of collaboration across disciplines, a pressing need for innovative solutions to complex problems and the desire to provide students with a more holistic and integrated learning experience (Chrysostomou, 2004). Hence, interdisciplinary collaboration in music education can be explained as the collaboration and integration of different disciplines or fields of study within the context of music education. It involves the interaction and cooperation between music educators, musicians, and professionals, from other disciplines, such as psychology, sociology, neuroscience, business, technology, and dance, among others.

In music education, interdisciplinarity has been acknowledged as a means to enhance student engagement, stimulate creativity, and develop critical thinking skills (Overland, 2013). By connecting music to other fields of study, educators aim to develop diverse talents and promote imaginative solutions in students (Peretz et al., 2021). One of the key drivers of this trend toward interdisciplinarity in music education is the changing landscape of the modern classroom. With the integration of technology and digital media into every aspect of our lives, students today are more likely than ever before to approach learning from multiple angles and perspectives (Gouzouasis and Bakan, 2011; Bennett, 2016; O’Leary, 2020).

However, despite the growing presence of interdisciplinarity in music education, there are still significant gaps in our understanding of the current landscape of collaboration formats. Previous studies have primarily focused on specific programs or partnerships within limited geographic areas, resulting in a lack of cross-regional comparative data on interdisciplinary activities and research productivity (Carfoot et al., 2017). Besides, quantitative mapping of collaborative initiatives across institutions has been minimal, and there is a scarcity of multidimensional measures to assess interdisciplinary engagement. Additionally, there is a lack of contextual qualitative insights that can help explain regional variations.

Therefore, this research aims to explore three study objectives:

(a) To analyze the current landscape of interdisciplinary activities related to music education at the top ranked universities in the world.
(b) Identify variations in interdisciplinary models of music students’ education, focus areas, and integration approaches through qualitative synthesis of institutional data. This provides an understanding on regional and institutional distinctions in the different interdisciplinary collaboration models, focus areas, and implementation approaches.
(c) Identify relationships between interdisciplinary initiatives and published research article and observe trends if any exists using the mixed methods data.

The rest of the paper is organized as follows. Section “2 Literature overview” provides a brief overview on prior and related studies on interdisciplinary music education. Section “3 Research methodology” explores the mixed method approach, analytical techniques and observed relationships. Section “4 Findings” provides the findings, followed by implications in sections “5 Implications of the study,” limitations and lastly conclusion.

## 2 Literature overview

### 2.1 Some prior studies on interdisciplinary music education models

Interdisciplinary models in music education have become an expanding area of scholarship as traditional boundaries get redefined for the 21st century. Various studies have examined collaborative formats spanning diverse domains to enrich teaching and learning (Luce, 2001; Gaunt and Westerlund, 2016). For instance, one study explored the interplay between the changing music industries, the needs of interdisciplinary music students, and the educational policies surrounding them (Ski-Berg, 2017). Another study focused on collaborative learning in higher music education, examining theoretical perspectives and research studies on expertise, collective creativity, shared knowledge practices, and power dynamics within collaborative learning (Gaunt and Westerlund, 2016). Additionally, the concept of student engagement in music education has gained attention, with a focus on preparing students for life beyond their education and exploring the merits and determinants of student engagement (Wang, 2021). The integration of interdisciplinary fields, such as music technology, into academic structures has also been examined, highlighting the challenges and opportunities in research, teaching, and administration (Boehm, 2005). Also, an interview-based approach discussed the importance of interdisciplinary dialog in music therapy and special music education, providing insights into the development of collaborative work between different music practices (Darrow and Tsiris, 2013).

One of the most widely studied interdisciplinary music education models is integrated arts education, which combines music with other art forms such as visual arts, dance, and drama. This model aims to develop students’ creativity, imagination, manual skills, and motor sensitivity (Budiwati, 2019; Avci, 2020). It emphasizes learning through experience and encourages students to actively participate in the learning process. By integrating music with other art forms, students are able to make connections between different disciplines and develop transversal and interdisciplinary integrated competencies. Studies have shown that technology-enhanced learning can improve students’ technical skills, creativity, and engagement [e.g., (Maba, 2020; Barneva et al., 2021)].

The studies referenced provide insights into interdisciplinary music education models and how they can promote deep learning outcomes while improving student engagement and motivation. However, limitations and challenges from these studies opens a gap for additional work. This includes exploring interdisciplinary music education programs across different global regions in more to understand distinguishing trends across these regions and how it relates to previous studies. Alongside standalone research projects and success stories, these publications could shed light on how incorporating interdisciplinarity affects the work of both students, instructors, and researchers.

### 2.2 Impacts of interdisciplinary collaborations

Interdisciplinary collaborations in music education have demonstrated significant positive outcomes and impacts on students, faculty, institutions, and the music landscape in general. Research findings indicate that these collaborations have a range of benefits. For example, students engaging in interdisciplinary collaborations have shown an increased level of reflexivity, enabling them to fostering a deeper understanding of their role in constructing perspectives on Otherness (Karlsen, 2021).

Interdisciplinary interactions between music education and history have also yielded positive effects. These interactions have influenced various aspects of music education, such as shaping musical preferences in classical music of the 20th century (Žnidaršič, 2021). Furthermore, interdisciplinary communication in pianists’ education has been found to contribute to the formation of vocational and pedagogical skills, as well as enhancing pianist skills (Lysenko et al., 2021). Collaborations between music and art education have facilitated hands-on activities for students, leading to the development of creativity skills (Martyniv et al., 2021). These interdisciplinary collaborations have been instrumental in the advancement of reflective, ethically engaged, and diversity-aware music education practices (Timonen, 2021). These interdisciplinary interactions in music education, involving history, pianists’ education, and art education, have contributed to interdisciplinarity by fostering the integration of diverse fields, multiculturalism by incorporating various cultural perspectives, and collaboration by promoting cooperation and shared expertise among different disciplines.

The concepts of interdisciplinarity, multiculturalism, and collaboration are closely linked and mutually reinforcing. Multiculturalism involves recognizing and appreciating diverse cultural perspectives, which is essential in interdisciplinary contexts where different disciplines offer unique viewpoints. Collaboration, on the other hand, involves working together across disciplines, including cultural perspectives, to address complex issues and achieve innovative solutions. Multiculturalism is a consequence of interdisciplinarity as it promotes the integration of diverse cultural perspectives in interdisciplinary collaborations, while collaboration is a consequence of multiculturalism as it requires active engagement and cooperation among individuals from diverse cultural backgrounds to achieve interdisciplinary goals (Reich and Reich, 2006).

What is more, interdisciplinary collaborations in music education have also extended to the fields of technology and business, yielding significant positive outcomes and impacts. These collaborations have opened up new avenues for innovation and entrepreneurship in the music industry.

Moreover, integration of technology in music education has transformed the learning experience for students. By incorporating digital tools, software, and emerging technologies, students have gained opportunities to explore music production, composition, and performance in innovative ways. This interdisciplinary approach has nurtured their creativity, refined their musical skills, and equipped them with critical thinking and problem-solving abilities necessary for success in the digital age (Waddell and Williamon, 2019; Zhang, 2022).

Overall, these collaborations have paved the way to explore understand what common themes of collaboration exist across the board in higher education institutions that embrace interdisciplinarity.

### 2.3 Initiatives by some global institutions

Reviewing models adopted by prominent universities provides useful benchmarking insights, as such institutions are often pioneers in designing innovative curricula, adopting emerging technologies, and forming high-impact partnerships due to greater resources and capacities. Prominent conservatories like Juilliard have spearheaded new directions in performing arts education (Gupta, 2019). Besides, many universities and colleges have started to incorporate interdisciplinary approaches into their music education programs, recognizing the value of collaboration across different disciplines and the potential for innovation that it offers. Universities now offer courses that combine music with other disciplines such as technology, engineering, and business to promote interdisciplinary collaboration. Some of these include:

(a) Berklee College of Music–Music Production and Engineering: This program combines music with technology and engineering, focusing on music production, recording techniques, and audio engineering (Berklee College of Music, 2022).
(b) New York University–Music Business: This program combines music with business and entrepreneurship, providing students with knowledge and skills in music marketing, artist management, copyright law, and more (NYU Steinhardt, n.d.; New York University, 2022).
(c) University of Hong Kong–Department of Music: The department offers courses that combine music with other disciplines, such as music technology, music education, and music psychology. Students can gain interdisciplinary knowledge and skills in areas like recording technology, music cognition, and music pedagogy (University of Hong Kong, 2022).
(d) Shanghai Conservatory of Music–Music Technology: The conservatory offers programs in music technology that combine music with technology and engineering. Students can explore areas such as digital audio processing, music production, and sound design (Shanghai Conservatory of Music, 2022).
(e) Seoul National University–Interdisciplinary Program in Music and Technology: This program combines music with technology, offering courses in sound synthesis, computer music, and interactive music systems. Students have the opportunity to work on interdisciplinary projects and collaborations (Seoul National University, 2022).

## 3 Research methodology

This section explores how music education intersects with other disciplines across QS highly ranked universities in different regions of the world (see Table 1). To normalize the selection of universities, the QS ranking was selected as a benchmark to help categorize the institutions according to regions (Top Universities, 2023). The mixed methods approach uses both qualitative and quantitative data analysis to provide a holistic view. The qualitative content analysis offers explicit information on trends, including whether institutions offer interdisciplinary programs, case studies, and partnerships and identify themes across them. The quantitative analysis used in the analysis are mainly descriptive statistics. These are percentages, frequencies, means and standard deviations. The quantitative analysis provides complementary insights. It reveals what fraction or proportions of institutions offer these initiatives and how that percentage varies across regions. Quantifying publication tallies also allows for exploring variations between regions. Together, qualitative findings on structures and quantitative data on frequencies and outputs help identify patterns. This aids in drawing meaningful inferences about opportunities and challenges in cross-disciplinary collaboration involving music education globally. The mixed approach thus creates a more comprehensive understanding of real-world interdisciplinary landscapes and activities. Overview of the information collected are as follows:

(a) Number of institutions: The top five highly ranked institutions per region was considered based on the QS World University Rankings by Subject 2023: Performing Arts category. This is due to the absence of an independent music discipline only category. However, due to limited data on Latin America and Africa, these regions were excluded.
(b) Number of published articles: Count of published articles related to interdisciplinary music education, reflects the school/department’s contribution to interdisciplinary initiatives in the most current circumstance, between the years January 1, 2018 and July 31, 2023. Only published articles are considered for this study since they undergo more rigorous peer review process, has broader reach and readership and are more thorough in content. The consideration of published papers in this research serves as a measure of interrelated output based on interdisciplinary music education models. By analyzing the published research articles, the research aims to identify relationships between interdisciplinary initiatives and the scholarly output in the field. This analysis allows for the observation of any trends or patterns in research information sharing related to interdisciplinary collaborations in music education. Moreover, this serves as a foundation for further research on trends and patterns, as well as research and knowledge gaps in interdisciplinary music education models. However, in this research only the abstracts for the articles were reviewed based on the definition of interdisciplinary collaboration in music education as explained in section “1 Introduction.” This is because abstracts are summaries of research works which contain the research background, aims and objectives and significance, methodology and findings.
(c) Data source for collecting published articles—Scopus: Data on publications were collected from Scopus. Scopus is a widely recognized and reputable academic database that provides access to a diverse range of scholarly literature. It encompasses one of the largest abstracts, citation databases, indexing a vast collection of peer-reviewed journals, conference papers, and other scholarly documents from various disciplines (Scopus, 2023).
(d) Duration for publications—The duration for the publications considered is between 2018 and 2023 (July to be precise). This period of 4–5 years provides ample time to assess any publication where available.
(e) Keyword and filtering: Published articles were filtered based on the keyword “music,” the Boolean operator “OR,” “music education,” followed by the Boolean operator “AND,” then “affiliation”—name of institution. The keyword “interdisciplinary” was not considered because it limited the scope and returned very limited data for some institutions. This is because some research works were interdisciplinary yet did not use the keyword “interdisciplinary.” Other details are as follows: (i) Scope—documents category; (ii) Search within—Article title, Abstract, Keywords, Authors: this is for searching keywords like music or music education; and (iii) Affiliation.
(f) Interdisciplinary programs, courses, or majors: Interdisciplinary programs, courses, or majors combine the study of music with other disciplines to provide students with a broader understanding of music’s role in society and its connections to various fields.
(g) Case studies: Case studies, success stories, or exemplary projects involving interdisciplinary collaboration in music education which are explicitly indicated by the institutions. This indicates the prominence of successful collaborations. Based on the listed categories, the data on the case studies was collected from the websites of the mentioned institutions. The study focused on gathering information from the institutions’ websites where they explicitly highlighted and showcased cases as interdisciplinary case studies, success stories, and projects centered around interdisciplinary collaboration in music education.
(h) Collaborative events: Collaborative events like seminars, conferences, performances, or exhibitions that feature music programs and other disciplines.

**TABLE 1:** QS top five ranking universities per region (Top Universities, 2023).

Rank	Region	Institution	Country	Type of college/department considered	No. students enrolled in the institution/department (2022/2023)	Year when department or institution was founded
5	North America	The Juilliard School	New York City, United States	Music Department	950	1926
8		Curtis Institute of Music	Philadelphia, United States	The Curtis Institute of Music	302	1924
11		New York University (NYU)	New York City, United States	Department of Music and Performing Arts	–	1965
15		University of Rochester	Rochester, United States	Arthur Satz Department of Music	–	2020
24		Harvard University	Cambridge, United States	Department of Music	120	1875
1	Europe	Royal College of Music	London, United Kingdom	The Music Conservatory	750	1882
2		Conservatoire national supérieur de musique et de danse de Paris (CNSMDP)	Paris, France	The Music Conservatory	1300	1795
3		Royal Academy of Music	London, United Kingdom	The Music Conservatory	860	1822
4		Universität für Musik und darstellende Kunst Wien	Vienna, Austria	The Music Conservatory	3000	1817
6		Royal Conservatoire of Scotland –Formerly Royal Scottish Academy of Music and Drama	Glasgow, United Kingdom	Music Department	900	1847
51–100	Oceania	Edith Cowan University	Joondalup, Australia	Western Australian Academy of Performing Arts (WAAPA)- Music Category	–	1980
51–100		Griffith University	Nathan, Australia	Music and Performing Arts Department	–	–
51–100		Monash University	Melbourne, Australia	Sir Zelman Cowen School of Music and Performance	–	1965
51–100		The University of Auckland	Auckland, New Zealand	Faculty of Creative Arts and Industries -School of Music	–	–
51–100		The University of Melbourne	Parkville, Australia	Faculty of Fine Arts and Music	7000	2009
13	Asia	Hong Kong Academy for Performing Arts	Hong Kong, Hong Kong SAR, China	School of music	–	1984
21		UCSI University	Kuala Lumpur, Malaysia	Institute of music	770	1990
40		The Central Academy of Drama, China	Beijing, China (Mainland)	The Department of Musical Theatre	–	–
42		Korea National University of Arts	Seoul, South Korea	School of music	–	1993
48		National University of Singapore (NUS)	Singapore, Singapore	Yong Siew Toh Conservatory of Music	220	2003

(– means not disclosed or identified data).

Partnerships/Affiliations: Partnerships or affiliations with other departments or institutions promoting interdisciplinary education. This indicates the department’s external collaborations.

Next, data collected based on these defined fields are shown in Tables 3–6. Besides the research goals associated with these data are also clearly indicated in Table 2.

**TABLE 2:** Categories of data collected and associated research goals.

Data collected	Research goal
Number of institutions	To identify highly ranked institutions per region in the performing arts category as a measure of interrelated output in interdisciplinary music education models.
Number of published articles related to interdisciplinary music education between 2018 and 2023	To analyze the scholarly output and identify trends in research information sharing relationship to interdisciplinary collaborations in music education.
Data source: Scopus	To gather a wide range of scholarly literature and access a diverse collection of peer-reviewed journals and conference papers.
Duration for publications: 2018–2023	To assess the publications within a specific time frame for thorough analysis.
Keyword and filtering: “Music” and “Music Education” with institution affiliation	To filter and search for relevant articles based on keywords and institution affiliation.
Interdisciplinary programs, courses, or majors	To examine the combination of music with other disciplines in educational programs.
Case studies: successful interdisciplinary collaboration projects indicated by institutions	To highlight and analyze prominent case studies showcasing successful interdisciplinary collaborations in music education.
Collaborative events and partnerships/affiliations	To identify collaborative events and partnerships that promote interdisciplinary education and external collaborations within the department.
Example of interdisciplinary program/majors	To showcase specific examples of interdisciplinary programs and majors that combine the study of music with other disciplines, providing a broader understanding of music’s role in society and its connections to various fields.

### 3.1 Descriptive overview of interdisciplinary activities at the selected institutions

This section examines the university or institution’s website to gather data on interdisciplinary initiatives as indicated by the institutions. Research sections are reviewed for examples of interdisciplinary project if indicated. Program listings are also analyzed to identify any interdisciplinary majors or courses offered. Success stories, or exemplary projects involving interdisciplinary collaboration in music education are also noted. Interdisciplinary partnerships and events involving industry or academia are recorded. Those clearly described as interdisciplinary in nature based on surface-level website details are documented.

**A. The Case of North American Institutions:**

*The Juilliard School:*

(i) Collaborative events and partnerships: The Juilliard School has cross-collaboration through its interdisciplinary projects facilitated by Juilliard’s Creative Enterprise. These projects extend beyond traditional divisions and encompass technology, literature, visual art, and more. The Center for Innovation in the Arts serves as a hub for Juilliard’s interdisciplinary and technology-driven activities inaugural interdisciplinary performance series, featuring students from the Music, Dance, and Drama divisions. Juilliard has a partnership with the New York Philharmonic and the American Composers Forum (The Juilliard School, n.d.).
(ii) Example of interdisciplinary program/majors: Option for music students to take minor courses or execute independent or collaborative projects in dance or drama as part of the music degree.
(iii) Case study: Notably, acclaimed flutist, composer, and performer Nathalie Joachim attributes her diverse musical career to the interdisciplinary foundation she acquired at Juilliard (The Juilliard School, n.d.).

*Curtis Institute of Music:*

(i) Collaborative events and partnerships: The institute collaborates with external organizations such as 92Y to establish cooperative initiatives like “Curtis at 92Y,” promoting cross-institutional and interdisciplinary engagement and artistic exploration (Curtis Institute of Music, n.d.).

*New York University (NYU):*

(i) Collaborative events and partnerships: NYU’s Interdisciplinary Music and Performing Arts Professions Summer Programs offer diverse opportunities such as Music Business and Screen Scoring, Instrumental and Vocal Performance, Music Technology, and Dance Education. The university also organizes workshops and various projects to enhance interdisciplinary learning experiences. NYU’s partnership with Nashville in the field of Production and A&R in the Music Industry aligns with the intersection of music creativity and business, providing students with valuable studio and classroom experiences focused on talent evaluation, artist development, and record-making from a creative/production perspective (NYU Steinhardt, n.d.).
(ii) Example of interdisciplinary program/majors: NYU offers a range of programs that include Music Technology, Music Technology and Integrated Design and Media, Music Therapy, and Music Technology combined with Computer Science and Engineering.
(iii) Case study: The Music and Audio Research Lab (MARL) serves as a community for collaborative research and projects within NYU’s Music Education Program with an objective to promote learning from different disciplines.

*University of Rochester:*

(i) Collaborative events and partnerships: A formalized partnership between the Eastman Community Music School (ECMS) and The Strong National Museum of Play, expanding musical programming for children and seniors (University of Rochester, n.d.).
(ii) Example of interdisciplinary program/majors: Offers a combined BA/MA Program in Ethnomusicology, a unique collaboration between the Arthur Satz Department of Music and the Musicology Department at the Eastman School of Music.
(iii) Case study: One notable interdisciplinary project involves leveraging Artificial Intelligence (AI) for music production, which includes an education and outreach component aimed at empowering future musicians. This project brings together researchers from the University of Rochester’s Eastman School of Music, Warner School of Education and Human Development, Hajim School of Engineering and Applied Sciences, and School of Arts and Sciences, along with Northwestern University’s McCormick School of Engineering (University of Rochester, n.d.).

*Harvard University:*

(i) Collaborative events and partnerships: Harvard organize events such as the interdisciplinary conference on musical media, titled “Instruments, Interfaces, Infrastructures,” which further fosters cross-disciplinary dialog and exploration. Additionally, Harvard has formalized a partnership with Berklee College of Music (Harvard University Department of Music, n.d.).
(ii) Example of interdisciplinary program/majors: Harvard has formalized a partnership with Berklee College of Music, enabling students to pursue a 5-year dual degree program, earning a Bachelor of Arts (A.B.) from Harvard and a Master of Music (M.M.) or a Master of Arts (M.A.) from Berklee. Students can create their own interdisciplinary experiences by taking courses in different departments, collaborating with students from other departments on projects. For instance, in ethnomusicology—Ethnomusicology students are required to take courses in both musicology and offerings outside the department, promoting interdisciplinary perspectives (Harvard University Department of Music, n.d.).
(iii) Case study: Exploration of Harvard and Berklee dual degree program on music education.

General overview of the above listed information are summarized in Table 3.

**TABLE 3:** Performance of music interdisciplinarity in North American Institutions.

University	Interdisciplinary programs/majors	Case studies	Collaborative events	Partnerships	Publications (2018–2023)
The Juilliard School	Yes	Yes	Yes	Yes	6
Curtis Institute of Music	–	–	Yes	Yes	2
New York University (NYU)	Yes	Yes	Yes	Yes	9
University of Rochester	Yes	Yes	Yes	Yes	–
Harvard University	Yes	Yes	Yes	Yes	12
Total parameters identified for a region	4	4	5	5	
% parameters identified for a region	80%	80%	100%	100%	

Yes = a minimum of one does exist or available and is clearly indicated.

(–) = not clearly indicated or cannot be confirmed to exist.

**B. The Case of European Institutions:**

*Royal College of Music*:

(i) Collaborative events and partnerships: The Royal College of Music offers studentships that include cross-partnership training courses, interdisciplinary collaborations, placements, and research opportunities with non-HE industry partners such as the Museum of London, National Archives, Google, Victoria and Albert Museum, and Wellcome Trust. Example of interdisciplinary program/majors.
(ii) Example of interdisciplinary program/majors: Interdisciplinary studies are available in areas like material culture and organology and performance science.
(iii) Case study: The school engages in projects like “Music, Migration and Mobility,” which explores the significance of migration for music (Royal College of Music, n.d.).

*Conservatoire national supérieur de musique et de danse de Paris (CNSMDP):*

(i) Collaborative events and partnerships: Collaborations range from MDW University in Vienna to the Taiwan National University of the Arts, New Music Dublin, Shanghai Percussion Festival, Sistema Greece, and socio-educational foundations in Latin America (Conservatoire de Paris, n.d.).
(ii) Example of interdisciplinary program/majors: CNSMDP offers “Sciences, Arts et Création” (Science, Arts, and Creation) program, which aims to bridge the gap between scientific research and artistic creation.

*Royal Academy of Music:*

(i) Collaborative events and partnerships: The academy organize seminars, workshops, and courses to develop core artist skills, self-knowledge, and professional awareness. Students benefit from a stimulating curriculum, a wide range of concerts and events, and mentorship from legendary artists. The academy also invests in bursaries, educational projects, collaborations, and industry partnerships (Royal Academy of Music, n.d.).
(ii) Example of interdisciplinary program/majors: They provide a range of undergraduate and postgraduate courses that allow students to combine different disciplines within music or across multiple areas of study. For instance, self-promotion, marketing and music business.

*Universität für Musik und darstellende Kunst Wien:*

(i) Collaborative events and partnerships: MUK develop services in informal learning, community music, and music communication in cooperation with non-university institutions (Musik und Kunst Privatuniversität der Stadt Wien, n.d.).
(ii) Example of interdisciplinary program/majors: Some of the interdisciplinary programs offered include Music and Media, Music and Theater, and Music and Psychology.

*Royal Conservatoire of Scotland:*

(i) Collaborative events and partnerships: The conservatoire promote interdisciplinary collaborations through initiatives like Science, Humanities, and Arts Research Exchange (SHARE). They also facilitate concerts based on collaborations between composers, performers, and research students, fostering professional partnerships from the beginning of students’ studies (Royal Conservatoire of Scotland, n.d.).
(ii) Example of interdisciplinary program/majors: Offers interdisciplinary studies with music. For example, the study of film and television production with music composition and sound design. Also is Bachelor of Education (Music) with Honors, which prepares musicians for teaching music in schools. The degree includes integrated school placements.
(iii) Case study: Under the subsection of alumni case studies on the website of Royal Conservatoire of Scotland, it listed some individuals who were successful using the approach of interdisciplinarity and serves as case study for students (Royal Conservatoire of Scotland, n.d.).

General overview of the above listed information are summarized in Table 4.

**TABLE 4:** Performance of music interdisciplinarity in European Institutions.

University	Interdisciplinary programs/majors	Case studies	Collaborative events	Partnerships	Publications (2018–2023)
Royal College of Music	Yes	Yes	Yes	Yes	186
Conservatoire national supérieur de musique et de danse de Paris (CNSMDP)	Yes	–	Yes	Yes	1
Royal Academy of Music	Yes	–	Yes	Yes	133
Universität für Musik und darstellende Kunst Wien	Yes	–	Yes	Yes	4
Royal Conservatoire of Scotland	Yes	Yes	Yes	Yes	13
Total parameters identified for a region	5	2	5	5	
% parameters identified for a region	100	40%	100%	100%	

Yes = a minimum of one does exist or available and is clearly indicated.

(–) = not clearly indicated or cannot be confirmed to exist.

**C. The Case of Oceania’s Institutions**

*Edith Cowan University (ECU):*

(i) Collaborative events and partnerships: Interdisciplinary events organized by staff and students per year in collaboration with community partners. Besides, students can pursue highly specialist training in areas both inside and outside of their chosen major with departmental partners.
(ii) Example of interdisciplinary program/majors: Bachelor of Music Education—combines music performance and music education, preparing students for a career as music teachers.
(iii) Case study: The university’s staff and postgraduate students at the Western Australian Academy of Performing Arts (WAAPA) are actively involved in interdisciplinary projects across various disciplines merging music and theatre. These projects aim to explore artistic practices, past and present, and address questions related to why, what, how, who, when, and where (Edith Cowan University, n.d.).

*Griffith University:*

(i) Collaborative events and partnerships: The university organizes conferences, such as the one on designing human-computer interfaces and interactions for musical performance called NIME (New Interfaces for Musical Expression). NIME brings together researchers and practitioners for lectures, installations, concerts, demonstrations, and workshops. Additionally, Griffith University’s project “Sustainable Futures for Music Cultures” aims to empower communities worldwide to build musical futures on their own terms (Griffith University, n.d.).
(ii) Example of interdisciplinary program/majors: Bachelor of Music Education: Offer programs in music and theatre, pedagogy and music education and music and technology.
(iii) Case study: Griffith Centre for Interdisciplinary Research (GCIR) and the Griffith Centre for Creative Arts Research (GCCAR) do support research that focuses on music: Promoting health and wellbeing through participatory music.

*Monash University:*

(i) Collaborative events and partnerships: The Sir Zelman Cowen School of Music and Performance offers opportunities to collaborate with professional musicians, performers, and researchers working across classical, electronic, experimental, jazz, interdisciplinary, popular music and critical performance studies (Monash University, n.d.).
(ii) Example of interdisciplinary program/majors: The university offers programs like composition and music technology, theatre, performance and music (Monash University, n.d.).

*The University of Auckland:*

(i) Collaborative events and partnerships: They present their research in various settings, including art galleries, professional conventions, dance stages, and concert platforms to serve learning opportunities for students from different fields. Partnerships with organizations like CJC Creative Jazz Club Aotearoa and international institutions like the Shanghai Conservatory of Music and Royal College of Music provide performance and interdisciplinary collaboration opportunities for students through videoconferencing projects (University of Auckland, n.d.).
(ii) Example of interdisciplinary program/majors: There conjoint degrees like Bachelor of Advanced Science (Honors)/Bachelor of Music, Bachelor of Commerce/Bachelor of Music, Bachelor of Design/Bachelor of Music, Bachelor of Engineering (Honors)/Bachelor of Music and many others that offer interdisciplinarity programs.
(iii) Case study: The university’s staff and postgraduate students actively engage in interdisciplinary research projects, collaborating with external experts and professionals.

*The University of Melbourne:*

(i) Collaborative events and partnerships: The university’s Music, Mind and Wellbeing initiative combines neuroscience with music and social wellbeing, collaborating with partners from music, science, health, education, and industry sectors. Partnerships with leading industry and event organizations like the Australian World Orchestra, Impact Australia, Australian Broadcasting Company (ABC), and Melbourne International Film Festival (MIFF) further enrich the university’s interdisciplinary engagement (University of Melbourne, n.d.).
(ii) Example of interdisciplinary program/majors: The University of Melbourne offers interdisciplinary courses like the Master of Music Therapy, specifically designed for those pursuing careers as music therapists. The program covers theoretical approaches to practice, performance teaching, ethnomusicology, and more, preparing students for diverse settings such as hospitals, special schools, aged care facilities, and community health programs.
(iii) Case study: Music, Mind and Wellbeing initiative combined neuroscience with music and social wellbeing.

General overview of the above listed information are summarized in Table 5.

**TABLE 5:** Performance of music interdisciplinarity in Oceania’s Institutions.

University	Interdisciplinary programs/majors	Case studies	Collaborative events	Partnerships	Publications (2018–2023)
Edith Cowan University	Yes	Yes	–	Yes	9
Griffith University	Yes	Yes	Yes	Yes	1
Monash University	Yes	–	Yes	Yes	6
The University of Auckland	Yes	Yes	Yes	Yes	–
The University of Melbourne	Yes	Yes	Yes	Yes	61
Total parameters identified for a region	5	4	4	5	
% parameters identified for a region	100	80%	80%	100%	

Yes = a minimum of one does exist or available and clearly indicated.

(–) = not clearly indicated or cannot be confirmed to exist.

**D. The Case of Asian Institutions**

*Hong Kong Academy for Performing Arts:*

(i) Collaborative events and partnerships: Music forums, with international artists, with over 100 artists visiting each year to hold masterclasses, offer individual tuition, or conduct concerts on varying themes and topics (The Hong Kong Academy for Performing Arts, n.d.).
(ii) Example of interdisciplinary program/majors: The school offers required courses such as Collaborative and Interdisciplinary Practice for Musicians, as well as elective courses like Arts Facility Management, Creative Industries, Arts Management, Collaborative Processes in the Performing Arts, and Wellness and Performing Arts.

*UCSI University:*

(i) Collaborative events and partnerships: The Institute of Music organizes events such as guest lectures, public talks, and masterclasses on various topics, including creativity and sustainable development (UCSI University, n.d.).
(ii) Example of interdisciplinary program/majors: Master of Music (Performance Studies) program–students engage in both solo and collaborative performances while also developing critical inquiry and research skills through a secondary specialization in teaching, research, or community music.

*The Central Academy of Drama, China*:

(i) Collaborative events and partnerships: The department has established close relationships with several renowned musical theater companies and teaching institutions, serving as a base for musical theater actor training, research, and practice in China (Central Academy of Drama, n.d.).
(ii) Example of interdisciplinary program/majors: The Department of Musical Theatre at the Central Academy of Drama offers courses that merge elements from both theater and music.

*Korea National University of Arts–School of Music:*

(i) Collaborative events and partnerships: The institute also hosts industry-related workshops, academic forums, and music technology concerts (Korea National University of Arts, n.d.).

Example of interdisciplinary program/majors: Offers Interdisciplinary Music Studies for both bachelor’s and master’s level.

(ii) Case study: The Computer Music Center undertakes research on music and sounds using computers, including projects related to virtual reality, video music, and sound work.

*National University of Singapore (NUS):*

(i) Collaborative events and partnerships: Collaborations between NUS and partner schools such as the Haute école de musique Genève (Switzerland), Sibelius Academy of the University of the Arts Helsinki (Finland), and Yong Siew Toh Conservatory of Music (Singapore) as part of the ConNext partnership (Yong Siew Toh Conservatory of Music, n.d.).
(ii) Example of interdisciplinary program/majors: NUS offers interdisciplinary programs such as the Master of Music Leadership, which emphasizes entrepreneurship, innovation, and equips graduates with the skills to navigate the rapidly changing music profession in the digital age.
(iii) Case study: Music and health research center at NUS, is the first dedicated research center in Southeast Asia focused on leveraging the efficacy of music for health and wellbeing.

General overview of the above listed information on whether or not these interdisciplinary activities are present are summarized in Table 6.

**TABLE 6:** Performance of music interdisciplinarity in Asian Institutions.

University	Interdisciplinary programs/majors	Case studies	Collaborative events	Partnerships (I/A)	Publications (2018–2023)
Hong Kong Academy for performing Arts	Yes	–	Yes	Yes	1
UCSI University-Institute of music	Yes	–	Yes	Yes	2
The Central Academy of Drama, China	Yes	–	Yes	Yes	1
Korea National University of Arts	Yes	Yes	Yes	Yes	1
National University of Singapore	Yes	Yes	Yes	Yes	14
Total parameters identified for a region	5	2	5	5	
% parameters identified for a region	100	40%	100%	100%	

Yes = a minimum of one does exist or available and clearly indicated.

(–) = not clearly indicated or cannot be confirmed to exist.

### 3.2 Quantitative analysis on regional performance

The Table 3 provides a proportion-based analysis for the information collected, In North America, 80% of the institutions offer “Interdisciplinary Programs/Majors/courses,” indicating a majority of the institutions in North America embrace interdisciplinary education. A total of 80% of the institutions indicate some “Case Studies.” All institutions in North America organize “Collaborative Events,” representing 100% occurrence. Additionally, 100% of the institutions establish “Partnerships” highlighting the significance of collaborative relationships in promoting interdisciplinary initiatives (see Table 3).

In Europe, “Interdisciplinary Programs/Majors” are available in all five institutions analyzed, representing 100% of the institutions. “Case Studies are identified in two out of the five institutions analyzed,” representing 40% of the institutions. All five institutions in Europe organize “Collaborative Events,” indicating a 100% occurrence. Similarly, “Partnerships” are established in all five institutions, representing a 100% occurrence (see Table 4).

In Oceania, all five institutions offer “Interdisciplinary Programs/Majors.” Furthermore, 80% of the institutions indicate “Case Studies.” Additionally, four out of the five institutions organize “Collaborative Events” representing 80% occurrence. Moreover, all five institutions establish “Partnerships,” reflecting a 100% occurrence (refer to Table 5).

In Asia, all five institutions analyzed offer “Interdisciplinary Programs/Majors.” However, the prevalence of “Case Studies” is relatively low, with only two out of the five institutions indicating case studies, representing a 40% occurrence. On the other hand, “Collaborative Events” are widespread, present in all five institutions. Additionally, all institutions in Asia establish “Partnerships,” emphasizing a focus on fostering collaborative relationships (see Table 6).

The data reveals substantial differences in interdisciplinary music education research across regions. Europe significantly outpaced others in total publications from 2018 to 2023 with 337 articles. Oceania followed with 77, while North America and Asia published far fewer at 29 and 19, respectively. This dominance is also reflected in Europe’s highest average articles per institution of 67.4 among the five universities observed. North America and Oceania averaged 5.8 and 15.4, respectively, while Asia had the lowest at 3.8 articles. Notably, the standard deviations offer insight into output variability within each region. Europe’s sizable standard deviation of 77.1 indicates high variations between institutional productivities relative to its mean. Smaller standard deviations between 4.4 and 23 for other regions imply their university outputs were more uniform on average (see Table 7).

**TABLE 7:** Statistics on published articles per region.

Region	Number of institutions covered (xi)	Total number of articles published (N)	Average number of articles published per region (μ = N/xi)	Standard deviation(σ)
North America	5	29	5.8	4.4
Europe	5	337	67.4	77.1
Oceania	5	77	15.4	23.0
Asia	5	19	3.8	5.1

### 3.3 Qualitative content analysis

The qualitative review reveals several common interdisciplinary approaches across regions, along with some notable distinctions. Formal interdisciplinary degrees are widely offered combining music with fields like business, technology, therapy and more. A prominent theme across North American institutions is their technology-driven approaches to fostering interdisciplinarity in music. Juilliard, NYU and University of Rochester showcase centers, projects and partnerships leveraging technologies like music production software, audio engineering and artificial intelligence. Case studies from these schools highlight career applications of such technology-integrated training.

Meanwhile, other regions emphasize interdisciplinarity through diverse means. European programs blend music with related domains in fields such as material culture and science. Collaborations span placements, research and academic-industry initiatives. Royal College of Music exemplifies partnerships enriching music studies. Oceania strengths include inter-institutional collaborations and community involvement through performance projects. Griffith University’s conference highlights experience design innovations.

While technology does not dominate, some Asian schools integrate it strategically. Korea National University hosts tech-focused events. NUS facilitates international courses expanding technical fluency. UCSI’s performance program nurtures research skills transferable to technology.

Across all regions, common themes include extensive collaborative events, diverse external partnerships and interdisciplinary curricula merging music with other arts or non-art subjects.

### 3.4 Interrelationships between interdisciplinary initiatives and publications

In North America, The Juilliard School had initiatives in all 4 categories (Interdisciplinary programs/majors/courses, case studies, collaborative events, and partnerships) and published 6 interdisciplinary articles, similar to NYU which also had all initiatives but published 9. Curtis had 2 initiatives and 2 publications. Though University of Rochester had initiatives in all categories like Juilliard and NYU, it did not publish any articles. Harvard, with initiatives in all categories, published the most at 12 articles. Thus, the number of initiatives does not seem to definitively correlate with higher publications in this region.

In Europe, Royal College of Music had initiatives in all categories and published the most at 186 articles. Royal Academy of Music had 3 out 4 initiatives and published 133. Despite initiatives in all categories except case studies, CNSMDP only published 1 article. Similarly, Universität für Musik published just 4 despite initiatives in most categories. Royal Conservatoire of Scotland’s balanced initiatives corresponded with 13 publications. More initiatives did not necessarily translate to more publications.

Across the region, Oceania, The University of Melbourne stood out with initiatives in all categories and publishing 61 articles, the highest output. Monash had 3 initiatives and published 6 papers. Edith Cowan had 3 of 4 initiatives and published 9 articles. Despite initiatives present, Griffith and The University of Auckland published the least or none. A higher percentage of initiatives did not necessarily correlate with more peer-reviewed publications on interdisciplinarity.

Across Asian institutions, National University of Singapore had full initiatives and published most at 14 articles. Despite Korea National University of Arts also having initiatives in all areas, it published just one article. Hong Kong Academy for Performing Arts, UCSI University-Institute of Music and The Central Academy of Drama, China each had initiatives in 3 of 4 categories but published one, two and one articles, respectively. More initiatives did not correlate with greater publications, as initiatives alone did not determine higher research outputs.

## 4 Findings

Qualitative findings show shared priorities on application and collaboration through industry partnerships, community enrichment, and formal interdisciplinary degrees, but also distinct strategic models–North American schools prioritize technology-enabled innovation while European ones emphasize research-led conservatory-centered interdisciplinarity. Curricular integration also differs, with Europe offering tailored elective packages and North America integrating diverse disciplines within programs. Though aggregate regional data shows a directional link between higher initiative frequencies and publications, specific institutional analysis reveals inconsistencies, implying that productivity depends on factors beyond activity occurrences alone, likely resources and prioritization. The quantitative analysis reveals Europe’s primacy in interdisciplinary music education research output, publishing 337 articles from 2018 to 2023 vs. 29 in North America, 77 in Oceania, and 19 in Asia. Europe also leads in average publications per institution at 67.4. Frequencies of interdisciplinary programs and events are high across most regions, but case studies are more prevalent in North America (80%) and Oceania (80%) than Europe (40%) and Asia (40%). Partnerships are ubiquitous (100% occurrence). Therefore, the quantitative data demonstrates Europe’s research dominance but variations in activities, while qualitative insights reveal both common ground and strategic distinctions across regions.

In summary, the analysis reveals that North American institutions prioritize technology-driven interdisciplinary approaches. For instance, the University of Rochester partners with multiple departments to create an AI-powered music production project, exemplifying North America’s technology focus. The Juilliard School showcases interdisciplinary performance projects and industry partnerships. Curtis Institute of Music collaborates with external organizations on initiatives like “Curtis at 92Y.” New York University organizes interdisciplinary workshops and summer programs and partnerships with the music industry. Harvard University hosts interdisciplinary conferences and partnerships with Berklee College of Music. In Europe, the institutions emphasize research-based interdisciplinarity. For example, the Royal College of Music engages in migration-focused projects like “Music, Migration and Mobility” which explores the significance of migration for music. The Conservatoire National Supérieur de Musique et de Danse de Paris engages in a wide range of global collaborations on interdisciplinarity. The Royal Academy of Music organizes interdisciplinary seminars, workshops, and courses. The Universität für Musik und Darstellende Kunst Wien develops community music services on a variety of interdisciplinary subjects. The Royal Conservatoire of Scotland facilitates concerts and interdisciplinary research collaborations. In Oceania, the data reveals Australian universities balance industry and community partnerships. For instance, the University of Melbourne created the cross-sector Music, Mind and Wellbeing initiative combining music, neuroscience, and social wellbeing. This demonstrates Oceania’s focus on impact-driven partnerships. Other institutions like Edith Cowan University organize interdisciplinary events with community partners. Griffith University hosts conferences on human-computer music interactions. Monash University provides opportunities to collaborate with professional musicians on varying cases. The University of Auckland partners internationally for performance collaborations. While some technology integration was observed, partnerships and events were more pervasive in Asia. Korea National University of the Arts hosts workshops and music tech concerts. The National University of Singapore promotes global partnerships and collaborations while also leveraging the efficacy of music for health and wellbeing. The Central Academy of Drama, China promotes partnerships with musical theater companies. UCSI University organizes guest lectures and talks on the subject of interdisciplinarity in music education. The Hong Kong Academy for Performing Arts hosts masterclasses and music forums in partnership with invited guests. Hence, these pioneering findings expose the significant diversity and complexity inherent in global interdisciplinary music education.

## 5 Implications of the study

Interdisciplinary collaboration has become an imperative in 21st century music education to cultivate multifaceted skills and spur innovation. Thence, this research study reveals significant complexity and regional variations in current interdisciplinary practices. The analysis of top global institutions exposes contrasting models, focus areas and outcomes.

In Europe, a long tradition of conservatories and music academies drives a strong research orientation and formal interdisciplinary degrees. Yet North America emphasizes technology-led innovation through partnerships with media, AI and emerging sectors. Australia balances industry collaboration with community enrichment. Hence, interdisciplinary engagement must align with institutional strengths and local contexts. Uniform models cannot address diverse priorities.

Significantly, industry and community partnerships emerge as a consistent priority across regions. Real-world application with social impact is a shared goal of interdisciplinary music education. Experiential learning, performances, competitions and live events provide vibrant platforms to apply interdisciplinary knowledge. Hence, music institutions should actively coordinate with companies, NGOs, public venues and community organizations to provide exposure and networking opportunities. Such immersive experiences can catalyze students to develop meaningful ventures.

At the policy level, assessments and incentives should move beyond activity frequencies to gauge competencies developed through interdisciplinary learning. Partnerships should also be nurtured strategically to enrich curriculum, teaching practices and research. Moreover, funding schemes that connect academia with external stakeholders can stimulate innovation.

Critically, technology, business, therapy, and other complementary domains are now inextricable from music education. Customized interdisciplinary degrees, modules and certificates can equip graduates with integrated skill sets aligned to evolving market needs. However, forced uniformity may undermine successful region-specific models. Institutions must retain flexibility to play to their unique strengths while embracing interdisciplinarity’s immense potential to create multifaceted musicians, vibrant communities, and cutting-edge ventures.

Therefore, this research highlights the significant diversity in current interdisciplinary practices globally. While industry and community collaboration emerge as a shared priority, regional variations suggest localized approaches bear more fruit than one-size-fits-all policies. As music education evolves for the 21st century, interdisciplinarity is instrumental but must be nurtured as a creative, socially conscious process, not an end in itself.

## 6 Limitations and future works

While this study offers valuable insights, it has some limitations that provide avenues for future work. Due to lack of standardized category for music institution ranking, the study limited data sorting to QS ranking: Performing Arts category. This may be biased since some schools are more inclined to full music majors while others are integrated with other arts majors like drama. Translation for some universities may also limit the true nature of the variables under study. Also, some institutions probably do not use department affiliations but rather the university’s affiliations which makes it harder to filter. Besides, the analysis relies on a small sample of top institutions and draws exclusively from publicly available secondary data on university websites and databases. Besides, relying solely on Scopus database also offers some bias. To mitigate this potential bias, it would be advisable to complement the analysis with data from other reputable academic sources or databases that provide a broader coverage of scholarly literature in future research works.

## 7 Conclusion

This study sheds light on the complex landscape of interdisciplinary collaboration in global music education through an integrated mixed-methods approach. Our quantitative analysis of leading institutions across regions provides valuable empirical data highlighting Europe’s leadership in publications and adoption. It also shows variations like North America’s stronger focus on technology integration. Qualitative insights provide crucial context, such as Europe’s research orientation vs. Oceania’s industry ties. Examining relationships reveals multifaceted drivers of productivity, underlining that initiatives alone do not dictate outcomes. These evidence-based findings uncover significant diversity in interdisciplinary models and influences. This implies customized local approaches aligned with institutional contexts are paramount to nurturing diverse skills and sparking innovation. While industry and community partnerships offer common ground, successful implementation depends on capitalizing on strengths. As music education evolves for the 21st century, interdisciplinarity must be embraced creatively rather than as an end goal. This study thus provides a sound empirical foundation to guide policies and strategies that avoid one-size-fits-all thinking. Institutions globally need flexibility to blend local priorities with best practices worldwide. No single approach holds all the answers, but hybrid models leveraging technology, research, experiential learning and strategic partnerships demonstrate great potential. We hope these pioneering insights energize and empower music educators globally to creatively embrace interdisciplinarity’s possibilities for their contexts. Further exploration is still needed on this vitally important frontier.

## Data availability statement

The original contributions presented in the study are included in the article/supplementary material, further inquiries can be directed to the corresponding author.

## Author contributions

JS: Conceptualization, Data curation, Formal analysis, Investigation, Methodology, Project administration, Resources, Supervision, Validation, Visualization, Writing – original draft, Writing – review and editing. YS: Investigation, Writing – original draft. GZ: Conceptualization, Formal analysis, Investigation, Methodology, Resources, Supervision, Validation, Visualization, Writing – review and editing.
